# Musical participation and positive youth development in middle school

**DOI:** 10.3389/fpsyg.2022.1056542

**Published:** 2023-01-18

**Authors:** Beatriz Ilari, Eun Cho

**Affiliations:** ^1^Department of Music Teaching and Learning, University of Southern California, Los Angeles, CA, United States; ^2^Haskins Laboratories, Yale University, New Haven, CT, United States

**Keywords:** positive youth development, popular music education, middle school, extracurricular activities, adolescence, music

## Abstract

**Introduction:**

Music is central in the lives of adolescents. While listening is usually the most common form of engagement, many adolescents also learn music formally by participating in school-based and extracurricular programs. This study examined positive youth development (PYD), school connectedness (SC), and hopeful future expectations (HFE) in middle school students (*N* = 120) with four levels of musical participation in school-based and extracurricular music programs. Levels of participation were based on students’ engagement in different music programs, including the Virtual Middle School Music Enrichment (VMSME), a tuition-free, extracurricular program that focuses on popular music education and virtual learning. We also investigated student listening preferences, musical tuition, and daily instrumental practicing.

**Method:**

Study participants completed an anonymous, online survey that contained five self-report measures including the very-brief form of the PYD questionnaire, a scale of school connectedness, and a scale of HFE.

**Results:**

Findings revealed significant differences in PYD scores by grade and gender, and associations between levels of musical participation and competence, a PYD component. Liking music and participation in extracurricular activities predicted scores on SC, and starting formal music education before age 8 predicted scores in HFE. We also found VMSME students to stem from neighborhoods with lower HDI than students in the other study groups, which points to issues of access to formal music education.

**Discussion:**

Findings are discussed in light of earlier research on PYD, extracurricular activities in adolescence, the ubiquity and functions of music in adolescence, and deficit thinking in education.

## Introduction

From its early beginnings, research on adolescence has been rooted on perspectives of deficit. “Storm and stress,” “developmental disturbance,” and “crisis” are terms that were used in the past when referring to adolescence (see Lerner, 2007; Bowers et al., 2010; Shek et al., 2019). These terms have accompanied the construction of a pervasive view of adolescents as “problems to be managed” (Bowers et al., 2010, p. 720). In light of such a view, a wide range of prevention programs were developed over the years to target problems like depression, delinquency, antisocial behavior, and substance use (Lerner, 2007). This is understandable as it is probably easier to focus on what young people should avoid like violence and drugs, or “not experience” (e.g., mental health issues) than to reach an agreement on experiences and adolescent characteristics that might enhance their lives, and help them thrive (Bowers et al., 2010, p. 720). Deficit thinking often hinders our capacity to view the strengths of young people and their potential to contribute positively to their communities. As a dominant paradigm, deficit thinking not only shapes research, practice and policy, but may also fuel a mistrust in young people and, consequently, interfere with “the natural and inherent capacity of human collectives (e.g., cities) to be community” (Benson, 2007, p.33).

In the past years, a new approach to adolescent development emerged. Moving away from the dominant paradigm of adolescence as a time of deficit that had been so pervasive in earlier research in psychology, education, developmental science and public health, positive youth development (PYD) focuses on the strengths of young people and their potential to contribute to society (Bowers et al., 2010; Lerner et al., 2011a; Shek et al., 2019). PYD has been conceptualized in multiple ways. Different frameworks and conceptual models have emerged in the literature, all predicated on individual-context relations that promote positive development in youth (Lerner et al., 2011a). In this study, we adopted Lerner’s Five Cs Model of PYD as our main theoretical framework (Lerner et al., 2011b). This model is not only well supported by empirical work, but also has been applied to the design and evaluation of programs aimed at youth (Bowers et al., 2010; Lewin-Bizan et al., 2010).

## Theoretical underpinnings: The Five C’s Model of PYD

Grounded on developmental systems theories, the Five Cs Model of PYD posits that positive development happens when the strengths of youth, such as their potential for growth and brain plasticity, are aligned with developmental assets (Bowers et al., 2010). Developmental assets theory, in turn, is based on conceptions of the developing individual, the context in which this individual is situated, and the dynamic interactions between them (Benson, 2007). This theory also takes into account the saliency of developmental processes like identity construction and issues of self-evaluation, autonomy and meaning making in adolescence, and adopts a view of adolescents as agentic beings who act upon and impact their contexts (Benson, 2007). The theoretical construct of developmental assets, therefore, refers to a group of environmental and interpersonal strengths that are known to promote and enhance educational and health outcomes in childhood and adolescence (Benson, 2007).

Developmental assets can be divided into internal (i.e., those related to dispositions, skills, competencies and commitments) and external (i.e., those related to contextual and relational issues within communities). Internal assets include *commitment to learning* (e.g., achievement motivation, school engagement and attitudes toward school), *positive values* (e.g., caring, responsibility, honesty), *social competencies* (e.g., interpersonal and cultural competence, resilience), and a *positive identity* (e.g., sense of purpose, positive view of one’s future). External assets are *support systems* (e.g., family support, parental involvement in school, positive family communication, caring school climate), *empowerment* (e.g., safety, service to others, youth as resources), *boundaries and expectations* (e.g., family and school boundaries, adult role models, high expectations), and *constructive use of time* (e.g., creative activities, youth programs; see Benson, 2007, pp. 7–9).

The positive development that emerges from the alignment of these developmental assets with the strengths of adolescence have been operationalized by five key domains known as “Five 5Cs” (Lerner et al., 2005; Lerner, 2007). The Five Cs of PYD emerged from earlier empirical research in adolescence, and from experiences of and language used by practitioners working with young people when defining youth “who are thriving.” Competence, confidence, connection, character, and caring & compassion are the Five Cs of PYD (Lerner, 2007; Bowers et al., 2010). *Competence* refers to positive views of one’s actions in social (e.g., interpersonal skills), academic (e.g., school grades, test scores), cognitive (e.g., decision making and self-regulation) and vocational (e.g., career-related explorations and entrepreneurship) areas. *Confidence* is an internal sense of positive self-worth and self-efficacy. *Connection* relates to positive, reciprocal relationships with peers, family members, schools and communities. *Character* refers to one’s respect for social and cultural norms, integrity and a “moral compass,” as well as standards for correcting behaviors. As Park (2004) suggested, character strengths such as kindness, optimism, hope and social self-control function as a buffer against mental and physical health issues, helping youth thrive. Finally, *Caring & Compassion* refer to sympathy, empathy and prosociality toward others (Bowers et al., 2010). Development in these five domains over time may lead to contribution, a sixth domain. *Contribution* expands beyond the self, and is associated with family, community, and civil society (Acosta et al., 2021; Bowers et al., 2010). Adolescents who manifest the Five Cs over time are more likely to make contributions to society (i.e., the sixth C), and are also less likely to be on a life journey that is marked by risk and problem behavior (Bowers et al., 2010). While PYD does not postulate happiness as one of its goals, the development of the 5Cs is “consistent with the acquisition of habits that lead to happiness in the eudaimonic sense” (Romer and Hansen, 2021, p. 84).

### PYD, extracurricular activities and music participation

As noted, PYD has been used in research and as a framework to develop and evaluate programs aimed at young people (Bowers et al., 2010). PYD has also been associated with young people’s participation in organized activities, including extracurricular programs that take place during out-of-school time (Mueller et al., 2011; Ettekal and Agans, 2020; Sorsdahl et al., 2021). A large number of U.S. children and adolescents participate regularly in one or more forms of organized activities (Pew Research Center, 2015). The term organized activities has been adopted by some scholars when referring to regimented, extracurricular activities that occur outside the school curriculum such as afterschool and youth programs held in schools, churches, community centers and clubs (Bohnert et al., 2010). These programs typically center around athletics, artistic and academic areas, tend to be structured and led by adults, and are typically designed and delivered for youth of similar ages at regular, pre-scheduled times (Friedman, 2013).

Several studies have examined outcomes derived from participation in a wide range of extracurricular program offerings (e.g., Howie et al., 2010; Metsäpelto and Pulkkinen, 2014; Ilari et al., 2016; Zarobe and Bungay, 2017; Molinuevo et al, 2010). A closer examination of these works suggests that outcomes are associated with demographics, individual/person factors (e.g., motivations and dispositions, interests), peer relations, family influence, and community characteristics and affordances. Because participation in extracurricular activities is multifaceted, their outcomes are also said to be mediated by breadth (number and types of activities), intensity (time devoted to activities), duration (months or years of participation), consistency of participation (regularity of participation) and individual levels of engagement (e.g., emotional, social, physical, cognitive; see Busseri et al., 2006; Bohnert et al., 2010; Oberle et al., 2019).

A solid body of evidence exists to support the association between prolonged participation in extracurricular activities and positive developmental outcomes (e.g., Catalano et al., 2002; Mueller et al., 2011). Aside from developing specialized skills in areas like music, dance, sports and academics, sustained participation in extracurricular programs may enhance cognitive, motor and socioemotional skills in children and youth (Mahoney et al., 2005; Fredricks and Eccles, 2006; Metsäpelto and Pulkkinen, 2014; Hedemann and Frazier, 2017; Zarobe and Bungay, 2017; Ilari et al., 2019). This includes the reduction of boredom and antisocial behaviors, and an increase in self-control, self-esteem and empathy in young people (Bone et al., 2021). Participation in extracurricular programs has also been associated with educational attainment and college admission, civil engagement in adulthood, and social mobility of youth from underserved communities (Snellman et al., 2015). These outcomes have been partly explained in terms of the characteristics and affordances of specific programs, which may allow youth to develop initiative and leadership skills, grit, socioemotional skills, agency and a sense of belonging (Mueller et al., 2011; Snellman et al., 2015; Zarobe and Bungay, 2017).

Extracurricular music programs may be particularly suitable for PYD, given the associations between musical engagement and psychosocial and emotional wellbeing (Saarikallio and Erkkila, 2007; McFerran et al., 2019; OECD, 2021), self-regulation (Krause et al., 2021), executive functioning (Hennessy et al., 2019), and community building (Eerola and Eerola, 2014). To date, only a handful of studies have examined adolescent participation in extracurricular music programs through the lens of PYD, most of which through qualitative approaches (e.g., Barrett and Bond, 2015; Hickey, 2018; Hospital et al., 2018). Of the existing studies, none was found to center on a learning model that incorporates digital technologies, popular music, acoustic instruments, and group classes involving a diverse student body. Uncovering the impact of such programs is vital, given the centrality of popular music and digital technologies in adolescence (North and Hargreaves, 1999; Lamont and Hargreaves, 2018), recent calls for reform in school music education (Rinsema, 2017; Schmidt, 2017), and the disruptions caused by the COVID-19 pandemic to education, and their associated perceptions of learning deficit, particularly in high-needs schools located in underserved neighborhoods (Lehman et al., 2021).

### Schooling, music education, and adolescent wellbeing in times of disruption

The United States is recognized worldwide for having a strong tradition in school music education. However, pre-pandemic studies with nationally representative samples suggested that only a fraction of U.S. students (i.e., 21% in 2004 and 24% in 2013) actually participate in high school music programs, with higher participation from affluent students and those attending large schools (Elpus and Abril, 2011, 2019). A recent study focusing on musical participation of ethnically diverse, middle-school students from predominantly low-income backgrounds revealed similar results. Conducted in Miami, Florida, the study found that only 22% of the entire student body was participating in school music programs (Alegrado and Winsler, 2020). A common finding to these large sample studies is the low participation of students from less affluent backgrounds, and a much higher participation of white and wealthier students in school music programs. Due to the many disruptions caused by COVID-19, it is likely that participation in school music programs has decreased even more, especially in high-needs schools and in specific populations (e.g., students with learning disabilities). Likewise, participation in extracurricular music programs was also negatively affected by the pandemic (Ilari et al., 2022).

Aside from disruptions to schooling, extracurricular program participation and leisure (see Ettekal and Agans, 2020), the pandemic has also taken a toll on mental health, raising stress levels and depression in youth (Ellis et al., 2020; Magson et al., 2020). A challenge that lies ahead is engaging youth in learning programs that may foster positive development and strengthen their mental health and wellbeing. Although there is no reliable, comparable data on young people’s wellbeing across the world, a report by UNICEF (2020) suggests that a very large number of children and adolescents from 41 predominantly wealthy nations do not have good mental wellbeing, with the U.S. ranking 32nd in the list. Analogously, a recent report by the OECD (2021) found socioemotional skills to have taken a dip in students aged 10–15 years since the outbreak of COVID-19. These findings are troublesome.

At the same time, studies conducted during the lockdowns imposed by the pandemic showed connections between musical engagement and emotional wellbeing in adolescents and adults (e.g., Carlson et al., 2021). This is not completely surprising, given people’s uses of music as a technology of the self (DeNora, 2002), and the links between musical participation during adolescence and socioemotional development and wellbeing (Saarikallio et al., 2014). Adolescents not only listen to more music than any other age group (Bonneville-Roussy et al., 2013), but also develop identities around musical practices and preferences (North and Hargreaves, 1999; Campbell et al., 2007; Miranda, 2013). Pre-pandemic studies suggest a central role of music and arts programs–in and out of schools– in anxiety and depression reduction (Hedemann and Frazier, 2017), and in the promotion of resilience and wellbeing (Zarobe and Bungay, 2017) in young people. Participation in music programs has also been associated with school connectedness (Eccles and Barber, 1999; Eerola and Eerola, 2014), a key factor in adolescent mental health and academic success (Gamboa-Kroesen, 2019). It is possible, then, that participation in a music learning program that capitalizes on youth musical preferences and popular music (Bonneville-Roussy et al., 2013), digital affordances (Valkenburg and Piotrowski, 2017), and student agency (Saarikallio et al., 2020) might function as a protective factor during the critical years of middle school, especially in these nihilistic times. To this end, we designed the current study to explore the associations between different levels of participation in music and PYD, school connectedness, and hopes and beliefs about the future in adolescents. Our study focused on the Virtual Middle School Music Enrichment (VMSME) program, an innovative and accessible extracurricular music program that was designed in response to the educational challenges imposed by the pandemic.

### The VMSME program

The VMSME Program was launched in summer 2020 through a partnership between the Fender Play Foundation and one of the largest school districts in the United States. Drawing from earlier research in expert learning theory, biomechanics, psychomotor learning, habit-building and motivation, the VMSME program was designed following five main principles: (1) students are initially inspired by the songs they hear and want to learn; (2) learning something new motivates students and keeps them engaged; (3) learning how to play an instrument is a complex psychomotor skill that requires specialized instruction; (4) relatable teachers provide excellent lessons, and (5) students learn in multiple ways and, therefore, require personalized learning tools. In the VMSME program, students are involved in guided, active, “bite-size,” integrated and song-driven learning. Teachers work with students from the first step – how to hold an instrument – all the way through fundamental skills, chords, technique and playing a wide range of songs.

The VMSME program offers middle school students a unique music learning experience through the provision of musical instruments, access to a music learning app called Fender Play, and group online music lessons with credentialed teachers, all free of charge. Interested students sign up to receive instruments at their home for the program on a “first-come, first-serve” basis. Aside from being tuition-free, the VMSME program is also unique in that it focuses on popular music education delivered through an online platform. Weekly music classes are offered for mixed groups of students who attend different schools in the district; students from widely different neighborhoods meet regularly and learn together in the same virtual class. Classes are offered in guitar, electric guitar, electric bass and ukulele, with teachers taking advantage of digital “breakout rooms” to focus on both individual technique and small group activities. Students are also encouraged to perform with their peers, and to record and submit recordings of their playing to their teachers, and for virtual concerts. The Fender Play app includes a carefully-designed learning sequence, and students have many choices of songs to play in terms of musical genres and styles. The Fender Play app also helps with practicing and progress tracking, and includes a video library with tutorials and performances. Since its inception, the program has been made available to more than 15,000 middle school students in a large U.S. metropolis, with a recent expansion into an “in-person” ukulele program in partnership with Grammy award winners. This new program is held in a specific neighborhood for elementary school students, reaching a wider age group. It should be noted that the school district served by the VMSME program caters to a large number of Latino/a students (73.8% in 2022–2023, according to official data), one of the populations marked by low participation in school music education (see Elpus and Abril, 2019; Alegrado and Winsler, 2020).

## Purpose of the study

The purpose of this study was to examine PYD, school connectedness, and hopeful future expectations in middle school students with four levels of musical participation in school-based and extracurricular music programs, with a special emphasis on the VMSME program. The levels of participation were: (1) VMSME only, (2) VMSME and school music programs, (3) in- and out-of-school music programs but no VMSME; and (4) no participation. We also investigated PYD, SC, and HFE as a function of student grade level, reported gender, and socioeconomic background.

## Materials and methods

### Participants

One hundred and eighty-six middle-school students attending public schools in a large urban center participated in the online survey. Included in the final analysis were 120 students (59 males, 51 females, 4 non-binary, 6 prefer not to answer), who completed the entire survey. Students ranged in age from 11 to 14 years (Median^age^ = 12 years), and were attending 6th (*n* = 56), 7th (*n* = 33), and 8th (*n* = 30) grades. Most students were of Latino ethnicity (52%), along with White (11%), African-American (3%), Asian-American (11%), and mixed-race/other (27%). This is largely consistent with the racial/ethnic makeup of the school district (Fingertip Facts, 2022).

Participating students came from 52 different middle schools (out of a total of 77) located in multiple neighborhoods and cities served by the school district. We used the American Human Development Index (HDI) corresponding to the neighborhoods and cities where the schools were located as an estimate of student socioeconomic status. The American HDI is a composite score of human development that includes three dimensions: life expectancy, educational attainment and median earnings. HDI scores in our study ranged from 2.26 (very low) to 9.24 (very high), with a mean score of 5.19 (*SD* = 1.50). In 2021, the American HDI scores for the state and county where the study took place were 5.85 and 5.43, respectively.^1^

Consistent with earlier works on extracurricular program participation and human development (Bohnert et al., 2010), we adopted a person-centered approach (see Oberle et al., 2019) to sort study participants into groups. A person-centered approach takes into account “the ways in which multiple variables are configured within individuals rather than the relative standing of individuals on multiple variables” (Good et al., 2011, p. 539). Based on the combinations of types and levels of musical participation reported by our participants, four study groups were devised: (1) VMSME: Students participating exclusively in this program (*n* = 69); (2) COMB: Students enrolled in combinations of VMSME and one or more in-school music programs (*n* = 16); (3) INOUT: Students participating in one or more music programs, in- and/or out-of-schools, but not in VMSME (*n* = 32); and (4) NM: Students not participating in any music education program (*n* = 3). Hereinafter, we refer to these study groups, respectively, as VMSME, COMB, INOUT, and NM.

Over 97% of our sample (*n* = 117) played one or more musical instruments, with plucked string instruments (electric guitar, acoustic guitar and electric bass and ukulele) being the most popular (*n* = 101), followed by keyboards (*n* = 24), bowed strings (*n* = 20), woodwinds and brass (*n* = 17), and percussion (*n* = 5). Among all students enrolled in the VMSME program (*n* = 85), the acoustic guitar was played by the majority (54%), followed by the electric guitar (22%), electric bass (13%), and the ukulele (11%). A small number of students (24%) participated in school-based music programs, and these included band (*n* = 11), orchestra (*n* = 7), general music (*n* = 5), Mariachi (*n* = 3), music technology classes (*n* = 3), and other (e.g., keyboard classes, jazz, *n* = 8). A large number of students in the COMB (62.7%) and INOUT (50.1%) groups began learning a musical instrument before middle school, whereas about 70% of students in the VMSME group picked up their instruments in middle school. This suggests that the VMSME program was likely a first opportunity for many middle-school students to engage with instrumental music education.

### Measures

The following measures were used in our study:

- The PYD – very short form (PYD-VSF, see Geldhof et al., 2013) is an abbreviated, self-report with 17 items using a 5-point Likert scale (1 = strongly disagree, 5 = completely agree). Participants are asked to rate their agreement with statements like “All in all, I am glad that I am me” (confidence) and “I do very well in my class work at school” (competence). The PYD-VSF yields individual scores for each of the five components of PYD and a composite score. PYD-VSF© Tufts University 2022. All rights reserved. Used with permission.
- The out-of-school time (OST) structured activity (National Mentoring Resource Center, n.d.), is an abbreviated scale of student extracurricular activities in schools, community centers, religious centers and others. It contains 4 items, and asks respondents to report time spent in different types of extracurricular activities over the past 6 months.
- The school connectedness (SC) subscale from the Hemingway Measure of Adolescent Connectedness (Karcher, 2003), measures engagement, enjoyment and success in school. This subscale includes 6 statements (e.g., “I feel good about myself when I am at school”), and respondents are asked to state how true they are for them, using a 5-point scale (1 = not at all, 5 = very true).
- An adapted version of the Hopeful Future Expectations Scale (Institute for Applied Research in Youth Development, 2022) containing 4 questions about student expectations towards the future (e.g., chances of being safe) using a 5-point scale (1 = very low, 5=very high). HFE © Tufts University 2022. All rights reserved. Used with permission.
- A 12-item, researcher-developed demographic profile on age, gender, ethnicity, SES/school location, grade, musical background, musical preferences, and music education experiences, in and out of schools.

### Data analysis

Analyses were performed using SPSS Statistics 28.0. We began with a descriptive analysis to explore participants’ engagement in music and in different types of extracurricular activities (OST). Next, we examined if there were group differences in PYD, SC, HFE, and OST using independent-samples *t*-tests, one-way analyses of variance (ANOVAs), and linear regression models. For the analysis of OST and age at the commencement of formal music education, we utilized non-parametric tests (i.e., Kruskal–Wallis *H*-test, Mann–Whitney *u*-test) since the dependent variables were ordinal. With ANOVAs, in the cases where the assumption of homogeneity of variance was violated (as assessed by Levene’s test of homogeneity of variances, *p* < 0.05), we used the Welch ANOVA with the Games-Howell post-hoc test.

Multiple regressions were then performed to examine the variables that best predicted students’ PYD, SC, and HFE scores. Variables shown to have significant bivariate relationships with any of PYD, SC, and HFE from the preliminary analysis were entered as potential predictors in the regression models. Predictor variables that were categorical (i.e., grade, gender, and music participation) were dummy-coded. All assumptions for multiple regression were tested prior to the analysis. There was independence of residuals, as assessed by Durbin-Watson statistics with values between 1.69 and 2.23. The assumptions for linearity and homoscedasticity were met, as assessed by visual inspection of plots of studentized residuals versus unstandardized predicted values. The tolerance values used for checking multicollinearity between independent variables were above 0.8. SPSS Casewise Diagnostics detected one outlier whose standardized residual was slightly greater than 3 standard deviations in PYD Confidence, Caring, and Connection; however, we kept the outlier, as it had little effects, as seen when the analysis was run with and without the case.

## Results

### Students’ musical participation, preferences and liking

**Figure 1:**
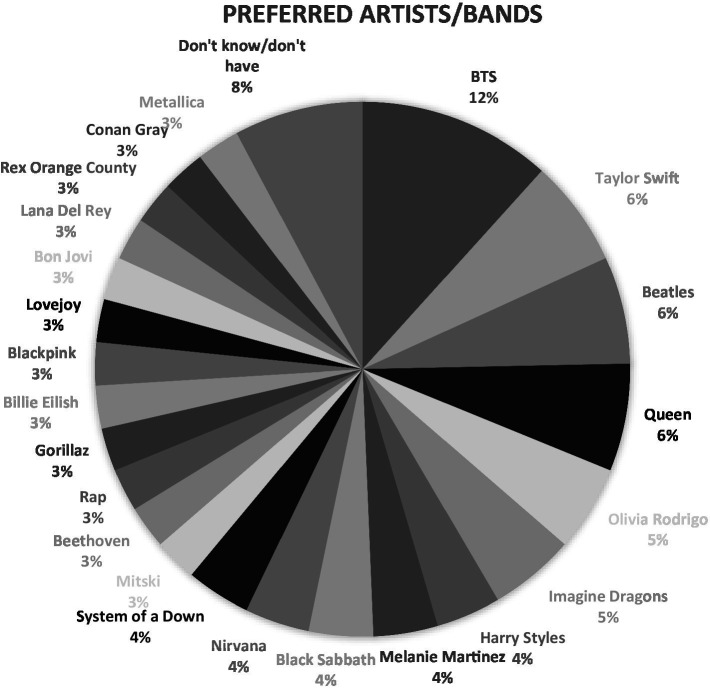
Students’ musical preferences.

Most participants (98%) were involved in one or more musical activities, in and out of schools, with a large number reporting that they practiced their instruments daily, but for less than an hour (77%). Only a small number of participants reported daily instrumental music practice for more than 2 h (4%). When asked to rate their overall liking for music using a scale of 1 to 5, with 5 being “I love music,” over 85% rated 4 or 5. Students’ high levels of musical engagement were also evident in the amount of time that they spent listening to music, as over 50% reported listening to music daily for more than 3 h. Their musical preferences and taste were very eclectic, involving a wide range of genres and styles, such as K-pop (e.g., BTS, Blackpink), rap, heavy metal, pop, indie, and Western classical music (see Figure 1). Interestingly, of a total of 152 responses, 20% were related to artists and bands from the 1990s or earlier (e.g., AC/DC, Beatles, Queen) which suggests some links to parents’/caregivers’ listening preferences and “reminiscence bump” (Krumhansl and Zupnick, 2013). Because the Fender Play app offered a variety of genres and styles for students to select from, including “older” pop tunes, it is also possible that VMSME students learned this repertoire through participation in the program.

### Participation in extracurricular activities

Most students in our sample participated in extracurricular activities offered at schools, outside of regular school hours (62%). Some students also participated in extracurricular activities in community spaces (e.g., Boys & Girls Club, YMCA; 35%), religious facilities (33%), and other settings (30%). A Kruskal–Wallis H test revealed no significant differences on levels of participation in extracurricular activities between study groups.

### PYD, SC, and HFE

We first calculated mean scores and standard deviations for PYD, SC, and HFE (Table 1) and compared them with earlier findings. Previous studies from different parts of the world (e.g., Malaysia: Abdul Kadir and Mohd, 2021; Philippines: Buenconsejo et al., 2022; Spain: Gomez-Baya et al., 2022; United States: Tyler et al., 2020) that have employed the PYD-VSF or PYD-SF (PYD short form; Geldhof et al., 2013) reported the following range of mean scores for its subcomponents: Competence, 3.19 to 4.16; Confidence, 3.64 to 4.62; Character, 3.85 to 4.16; Caring 4.07 to 4.45; Connection, 3.67 to 4.17. Mean scores from our study were comparable, given that our participants scored 3.52 (*SD* = 0.82) on Competence, 3.83 (*SD* = 0.91) on Confidence, 4.20 (*SD* = 0.57) on Character, 4.38 (0.61) on Caring, and 3.98 (*SD* = 0.64) on Connection.

**Table 1:** Means and standard deviations for PYD, SC, and HFE.

Variables		*n*	PYD						SC *M* (*SD*)	HFE *M* (*SD*)
			Overall *M* (*SD*)	Competence *M* (*SD*)	Confidence *M* (*SD*)	Character *M* (*SD*)	Caring *M* (*SD*)	Connection *M* (*SD*)		
Grade	6th	58	4.13 (0.41)	3.63 (0.75)	4.13 (0.71)	4.28 (0.45)	4.44 (0.55)	4.13 (0.50)	4.01 (0.41)	4.25 (0.60)
	7th	32	3.98 (0.41)	3.51 (0.71)	3.68 (0.84)	4.21 (0.51)	4.55 (0.46)	3.91 (0.55)	3.86 (0.41)	4.08 (0.57)
	8th	30	3.75 (0.72)	3.36 (0.97)	3.42 (1.12)	4.04 (0.78)	4.08 (0.76)	3.76 (0.87)	3.93 (0.44)	4.15 (0.59)
Gender	Male	59	4.00 (0.53)	3.63 (0.74)	3.97 (0.78)	4.12 (0.66)	4.30 (0.66)	3.97 (0.63)	3.91 (0.43)	4.15 (0.61)
	Female	51	4.06 (0.48)	3.44 (0.88)	3.84 (0.97)	4.33 (0.43)	4.52 (0.45)	4.10 (0.58)	4.06 (0.37)	4.27 (0.59)
	Other	10	3.59 (0.53)	3.40 (0.73)	2.97 (0.87)	4.03 (0.48)	4.13 (0.88)	3.38 (0.64)	3.80 (0.51)	3.93 (0.43)
Music participation	VMSME	68	3.94 (0.51)	3.38 (0.80)	3.75 (0.95)	4.20 (0.57)	4.32 (0.62)	3.96 (0.60)	3.92 (0.42)	4.06 (0.60)
	COMB	16	3.99 (0.82)	3.56 (1.03)	3.69 (1.13)	4.34 (0.77)	4.38 (0.82)	3.94 (1.06)	3.94 (0.43)	4.41 (0.52)
	INOUT	31	4.13 (0.34)	3.89 (0.51)	4.10 (0.62)	4.11 (0.44)	4.50 (0.49)	4.06 (0.48)	4.09 (0.42)	4.36 (0.54)
	NM	3	3.82 (0.47)	3.11 (0.96)	3.67 (1.33)	4.00 (0.50)	4.33 (0.58)	3.92 (0.38)	3.89 (0.38)	3.83 (0.14)
Age at the beginning of formal music training	Before 8	28	3.97 (0.46)	3.58 (0.77)	3.89 (0.84)	4.19 (0.56)	4.33 (0.58)	3.82 (0.63)	3.90 (0.42)	3.96 (0.56)
	8–11	56	4.03 (0.55)	3.61 (0.78)	3.89 (0.85)	4.21 (0.56)	4.37 (0.69)	4.02 (0.68)	4.00 (0.38)	4.30 (0.56)
	12 and over	34	3.95 (0.54)	3.36 (0.86)	3.69 (1.06)	4.16 (0.59)	4.42 (0.53)	4.03 (0.58)	3.96 (0.49)	4.20 (0.62)

Adapted with permission from the Institute for Applied Research in Youth Development, Tufts University, 2022. Available at: (https://sites.tufts.edu/iaryd/). **PYD-VSF, HFE © Tufts University 2022. All rights reserved. Used with permission.**

Next, we examined if any group differences existed on PYD, SC, and HFE due to demographic variables (i.e., grade, gender, HDI) and levels of participation in extracurricular activities (i.e., OST scores). A one-way Welch ANOVA revealed significant differences on Overall PYD (composite) scores, Welch’s *F*(2, 57.824) = 4.104, *p* = 0.022, *η*^2^ = 0.088; and on PYD components: *Confidence*, Welch’s *F*(2, 56.724) = 6.844, *p* = 0.002, *η*^2^ = 0.113; *Caring*, Welch’s *F*(2, 62.174) = 4.293, *p* = 0.018, *η*^2^ = 0.088; and *Connection*, Welch’s *F*(2, 56.866) = 3.278, *p* = 0.045, *η*^2^ = 0.059. A Games-Howell *post hoc* analysis showed that 6th graders had higher scores for *Confidence* than 7th graders (95% CI (0.03 to 0.88), *p* = 0.032), and also scored higher on Overall PYD (95% CI (0.03 to 0.72), *p* = 0.028) and *Confidence* (95% CI (0.17 to 1.25), *p* = 0.008) than 8th graders. Seventh graders scored higher on *Caring* (95% CI (0.08 to 0.86), *p* = 0.014) than 8th graders. In addition, we found significant differences for Overall PYD scores, *F*(2, 117) = 3.574, *p* = 0.031, *Confidence*, F(2, 117) = 5.689, *p* = 0.004, *η*^2^ = 0.089 and *Connection*, F(2, 117) = 5.808, p = 0.004, *η*^2^ = 0.095, between different gender groups. A Tukey *post hoc* analysis revealed that students who self-identified as non-binary or who chose not to report their gender scored significantly lower than students who identified as females on Overall PYD (95% CI (−0.89 to −0.52), *p* = 0.023) and *Connection* (95% CI (−1.13 to −0.22), *p* = 0.003), and lower than males on *Confidence* (95% CI (0.08 to 0.86), p = 0.014) and *Connection* (95% CI (−1.03 to −1.00), *p* = 0.015).

There were no significant differences on SC and HFE due to the demographic variables; however, linear regressions showed that levels of participation in extracurricular activities held at schools (outside of regular school hours) significantly predicted students’ scores of SC, *F*(1, 110) = 5.822, *p* = 0.017, adjusted *R*^2^ = 0.042, and HFE, *F*(1, 110) = 12.970, *p* < 0.001, adjusted *R*^2^ = 0.097. We found no significant relationships between HDI and PYD, SC, and HFE scores.

We also checked whether there were any differences on PYD, SC, and HFE scores due to music-related variables (i.e., levels of music participation, age at the beginning of formal music education, music liking, hours spent on music practicing, hours spent listening to music). Students who were participating in one or more musical programs, in and out of schools, were included in the analyses (*n* = 117). A significant difference was found for PYD *Competence*, Welch’s *F*(2, 37.155) = 5.160, *p* = 0.011, *η*^2^ = 0.066, and for HFE, *F*(2, 112) = 4.251, *p* = 0.017, *η*^2^ = 0.079. A Games-Howell *post hoc* analysis revealed that INOUT students rated themselves significantly higher for *Competence* than VMSME students (95% CI (0.12 to 0.79), *p* = 0.005). We also found a significant difference on HFE due to the age when students first began their formal music education, *F*(2, 112) = 3.180, *p* = 0.045, *η*^2^ = 0.053. Students who started to learn instrument(s) before age 8 scored higher than those who began at ages 8–11 years (95% CI (0.02 to 0.66), *p* = 0.036). Lastly, a linear regression revealed that how much students liked music significantly predicted their scores on SC, *F*(1, 114) = 4.659, *p* = 0.033, adjusted *R*^2^ = 0.031.

Additionally, an independent-samples t-test revealed that VMSME students tended to come from schools located in lower HDI neighborhoods, compared to COMB and INOUT students, *t*(114) = 2.086, *p* = 0.039. Furthermore, VMSME students tended to participate in fewer extracurricular activities at schools than their COMB and INOUT peers, *U* = 1146.5, *z* = −2.298, *p* = 0.022, *d* = −0.310, and also tended to begin formal music instruction at later ages (i.e., over 12 years), *U* = 2161.0, *z* = 2.829, *p* = 0.005, than COMB and INOUT students. It seems evident that the VMSME program has been effectively serving students who might not have access to music learning in schools through curricular and afterschool programs.

### Predicting PYD, SC, and HFE

Multiple regressions were performed to examine which variables might predict students’ PYD, SC, and HFE scores. Table 2 presents the unstandardized regression coefficients and standard errors of all regression models. Predictor variables included three music-related variables (i.e., music liking, hours spent on daily music practice, level of music participation), one extra-curricular activity-related variable (i.e., OST1), and two demographic variables (i.e., grade and gender) that showed significant bivariate relationships with any of the criterion variables from the preliminary analyses. Music participation, grade, and gender were dummy-coded, with VMSME students (music participation), 6th or 8th graders (grade), and female students or those who self-identified as non-binary and chose not to report their gender (gender) being the base variables.

**Table 2:** Multiple regression analyses of predictors of PYD, SC, and HFE.

Predictor variables	PYD				SC *B* (*SE*)	HFE *B* (*SE*)
	Competence *B* (*SE*)	Confidence *B* (*SE*)	Caring *B* (*SE*)	Connection *B* (*SE*)		
Music liking	0.21* (0.11)	−0.03 (0.12)	−0.05 (0.08)	0.14 (0.08)	0.08 (0.05)	0.08 (0.08)
Music practice	0.11 (0.09)	0.17 (0.10)	−0.03 (0.07)	0.14 (0.07)	0.07 (0.05)	0.06 (0.06)
^1^Music participation VMSME = 1	−0.36* (0.16)	−0.05 (0.18)	−0.02 (0.05)	0.07 (0.12)	−0.04 (0.08)	−0.23* (0.11)
^4^Extracurricular activity participation	0.02 (0.07)	0.07 (0.08)	−0.06 (0.12)	0.08 (0.05)	0.07* (0.03)	0.14** (0.05)
^**2**^**Grade**						
6th = 1	0.20 (0.15)	0.55* (0.17)		0.25* (0.12)	0.13 (0.08)	0.12 (0.11)
8th = 1			−0.42** (0.14)			
^**3**^**Sex**						
Female = 1	−0.22 (0.16)	−0.03 (0.18)	0.24* (0.12)		0.10 (0.08)	0.10 (0.11)
Other = 1				−0.49* (0.22)		
*R* ^2^	0.123	0.123	0.133	0.186	0.146	0.185
Adjusted *R*^2^	0.073	0.073	0.083	0.139	0.097	0.139
*N*	112	111	112	112	112	112

^1^Dummy-coded, VMSME was the base variable; ^2^Dummy-coded, 8th grade was the base variable for Caring while 6th grade was the base variable for other criterion variables. ^3^Dummy-coded, “Other” was the base variable for connection while female was the base variable for other criterion variables; ^4^Measured by OST1. *p < 0.05, **p < 0.01, ***p < 0.001.

In the analysis of PYD, the multiple regression model significantly predicted scores in four of its subcomponents, *Competence*, *F*(6, 105) = 2.459, *p* = 0.029, adjusted *R*^2^ = 0.073; *Confidence*, *F*(6, 105) = 2.450, *p* = 0.029, adjusted *R*^2^ = 0.073; *Caring*, *F*(6, 105) = 2.683, *p* = 0.018, adjusted *R*^2^ = 0.083; and *Connection*, *F*(6, 105) = 3.986, *p* = 0.001, adjusted *R*^2^ = 0.139. Three of the PYD sub-components, *Confidence*, *Caring*, and Connection, were significantly predicted by the demographic variables grade and/or gender. None of the music-related variables significantly predicted these PYD sub-components, except for *Competence* which was predicted by students’ level of music participation.

The multiple regression model also predicted scores for SC, *F*(6, 105) = 2.989, *p* = 0.010, adjusted *R*^2^ = 0.097. Participation in extracurricular activities measured by OST1 (*t* = 2.118, *p* = 0.037) was the significant predictor, with students with higher participation in extracurricular activities at schools feeling more connected to school than those who were not. Similarly, the regression model significantly predicted scores for HFE, *F*(6, 105) = 3.981, *p* = 0.001, adjusted *R*^2^ = 0.139. In addition to students’ participation in extracurricular activities (*t* = 2.998, *p* = 0.003), level of music participation also emerged as a significant predictor (*t* = −2.079, *p* = 0.040) for HFE scores. Specifically, COMB and INOUT students were likely to score higher on HFE than VMSME students.

## Discussion

In this study, we adopted a person-centered approach (see Oberle et al., 2019) to explore the associations between PYD, SC, and HFE in adolescents with different levels of participation in school-based and extracurricular music programs. We were particularly interested in the experiences of adolescents participating in the recently-created VMSME program, which focuses on popular music and online classes for mixed groups of students. We also investigated PYD, SC, and HFE as a function of grade level, gender, and socioeconomic background.

Contrary to earlier research that suggested an increase in PYD scores over time (e.g., Lewin-Bizan et al., 2010), we found younger students to show higher scores for some PYD components than their older peers. Sixth grade students scored higher for overall PYD than 8th graders, and also showed higher scores for *Confidence* than their 7th and 8th grade counterparts. Additionally, 7th grade students scored higher for overall PYD than 8th graders. These findings can be interpreted in at least three different ways. First, studies that found an increase in PYD scores usually adopted a longitudinal design (Lewin-Bizan et al., 2010), whereas we used a cross-sectional design. Therefore, it is possible that our results were impacted by the nature of our study. Second and on a related note, it is possible that the decrease in PYD scores by grade levels reflected different developmental trajectories of PYD, as seen in earlier longitudinal studies. Lewin-Bizan et al. (2010), found four distinct patterns for PYD scores in U.S. adolescents over time (i.e., decreasing, increasing-to-stable-moderate, increasing/decreasing, and increasing-to-stable-high). Similarly, in a study that adopted a person-centered approach, Good et al. (2011) found different patterns of stability and change in adolescent development over time, which reinforces the role of individual differences. Although the design of the present study does not allow one to make claims about developmental trajectories and stability/change in adolescent responses, we suspect that differences in PYD scores could have been due to these factors. It is possible that students in 8th grade who took part in our study were undergoing different developmental trajectories than their 6th and 7th grade peers, as reflected in their PYD ratings. Third, our study centered on the experiences of middle school students. Middle school is known to be a difficult time for students as they grapple with the transition from childhood to adolescence and new social pressures. It is during the middle school years that early adolescents work toward “fitting in.” In this search to find their niche, adolescents calibrate and organize their social identities (Graham, 2018). Unsurprisingly, the role of peers peaks in middle school, and so do vulnerability and peer victimization (Graham, 2018). Most 8th grade students in our study had spent a considerable time immersed in online learning due to the pandemic, with limited social interaction with peers. Perhaps their lower scores for overall PYD and Confidence reflected challenges to adapt to their first and final year of middle school upon returning to in-person learning.

Gender differences for Overall PYD and specific components that emerged in our data are also worthy of commentary, as they partially mirror earlier findings. For example, Chauveron et al. (2015), who studied Scottish 7th grade students, found males to score higher in *Confidence* and *Competence,* and females to score higher on *Character* and *Caring*. Zimmerman et al. (2008), who analyzed data from the longitudinal 4-H study, found females to score higher on overall PYD than males. Gomez-Baya et al. (2022), in turn, found Spanish males to score higher on *Confidence* and *Competence*. and females to show higher scores for *Connection, Caring,* and *Character*. Yet, a limitation of these studies was their focus on the gender binary. In our study, students were able to choose from multiple gender categories. Students who chose the categories “non-binary” and “prefer not to answer” were grouped together and found to score lower than females on overall PYD and C*onnection*, and lower than males on *Confidence* and *Connection.* While these findings are in agreement with earlier works that showed higher scores for *Confidence* in males (Chauveron et al., 2015; Gomez-Baya et al., 2022), and higher scores for overall PYD in females (Zimmerman et al., 2008), they are also of concern. Given recent findings on high levels of depression and suicidality rates in LGBTQ+ and non-gender conforming students (Rawlings and Espelage, 2020; Ancheta et al., 2021), it is central that future research using PYD as a framework move beyond the gender binary. Findings from these studies will not only contribute to our understanding of PYD in adolescence, but will also inform the development of programs and policy for all young people.

It was also interesting that students in the INOUT and COMB groups scored higher for *Competence* than VMSME students. *Competence* refers to positive views of one’s actions in social, academic, cognitive, and vocational areas (Bowers et al., 2010), including agentic and adaptive decision-making skills (Romer and Hansen, 2021). This finding does not come as a complete surprise if we consider the novelty of the VMSME program. Additionally, some participants in the INOUT and COMB groups were enrolled in private lessons and/or playing in small ensembles where there is comparatively more individual attention than in large group classes. Many of these participants also had more years of formal music education than their VMSME counterparts. The latter came from lower HDI areas and were attending fewer extracurricular programs in schools than their peers in the INOUT and COMB groups. Because intensity and breadth of extracurricular programs play a role in the development of positive attributes in youth (Busseri et al., 2006), it is possible that the differences in *Competence* scores between VMSME, and INOUT and COMB students were more reflective of socioeconomic issues and time in programs, rather than the characteristics of the music programs themselves. Future studies could disentangle these issues by examining the development of *Competence* and other PYD components in music students from the VMSME and other music programs using a longitudinal design, controlling for participation time, and program breadth and intensity.

By giving access to instruments and music classes for students from low HDI areas, a population that is often left out of school music programs (see Elpus and Abril, 2011; Alegrado and Winsler, 2020), the VMSME program contributed to the democratization of music education in the studied school district. This is also evidenced by the fact that INOUT students, who were participating in one or several music programs, showed higher ratings for *Competence* and HFE than the VMSME group. Participation in COMB and INOUT programs also predicted student scores on HFE, an adolescent strength that is theorized to partly assist with achieving future goals (Schmid et al., 2011). This finding not only reinforces the central role of VMSME as an enrichment program (and not a substitute for in-school music programs), but also hints at the idea that more time spent in music programs—in and out of schools—may contribute to the development of one’s hopeful expectations toward the future. We hypothesize that prolonged participation in music programs can, indeed, lead to enhanced and positive expectations toward the future, due to the many links between music learning, skill development, character building (Hallam and Cuadrado, 2018), and socioemotional development and emotional regulation (Saarikallio and Erkkila, 2007; McFerran et al., 2019; OECD, 2021). Formal music education has also been associated with the development of self-regulation (Krause et al., 2021), which is another predictor of PYD in adolescence (Schmid et al., 2011). Our finding that students who started their formal music education before age 8 scored higher on HFE supports this hypothesis.

Participation in extracurricular activities in schools predicted scores on School Connectedness. This is not surprising, given earlier research that links extracurricular activities to student positive experiences in schools (e.g., Eccles and Barber, 1999; Romer and Hansen, 2021). Music liking also predicted scores on SC, which could possibly be explained by student emotional regulation through music listening (Saarikallio and Erkkila, 2007; McFerran et al., 2019) and identity work (North and Hargreaves, 1999; DeNora, 2002; Miranda, 2013; Lamont and Hargreaves, 2018). Music has been defined as a “badge of identity” in adolescence (North and Hargreaves, 1999), with musical preferences playing significant roles in friend selection and group affiliation (Franken et al., 2017). Musical preferences have also been associated with externalizing behaviors in adolescence (Franken et al., 2017). It is possible that through the expression of their eclectic musical preferences (as seen in Figure 1), our participants found ways to express and regulate their emotions, and developed intrapersonal skills during the challenging years of middle school. That is, music may have served as a protective factor (see Miranda, 2013), helping to strengthen student confidence and connections with their schools. While this explanation is in line with earlier research (Eerola and Eerola, 2014), it could be further explored.

Finally, we would like to highlight the role of the VMSME program in promoting access to music education, and opportunities for middle-school students to experience diversity and inclusion. Aside from a focus on popular music, instrumental learning and the use of an educational app, a central characteristic of the program was the delivery of online music lessons by credentialed music teachers for mixed groups of students from different neighborhoods. This aspect of the program was not only praised by its music teachers and students, but is actually vital in our current context. Due to the pandemic, schools went on lockdown, depriving young people from physical and, in many cases, social contact with same-age peers for several months, at a time when peer groups are central (Graham, 2018). Additionally, public schools, particularly in urban areas, are often siloed. With the exception of occasional athletic, artistic or academic competitions, there are few opportunities for students from different schools to interact. By bringing together students from different neighborhoods, the VMSME may have also contributed to student social identity development. Middle school students have been shown to develop more complex social identities and more positive attitudes toward outgroups when they participate in extracurricular programs that are diverse (Graham, 2018). While this study did not investigate social identity development in the context of VMSME, this is another point for future exploration.

## Concluding remarks

In this study, we found students who participated in multiple forms of music education (including the VMSME program) and for longer periods of time to score high on *Competence* (a component of PYD) and on HFE. Participation in extracurricular activities in schools and liking music also predicted students’ SC. These findings add to the body of literature on the role of curricular and extracurricular music programs in adolescent development as they relate to the acquisition of specialized skills, emotional regulation and character building (Hallam and Cuadrado, 2018), and student positive connections to schools (Eccles and Barber, 1999; Eerola and Eerola, 2014; see also Weiss et al, 2017). Our findings also align with the idea that through music programs, adolescents may regulate and work through emotions and engage in identity work, developing positive views of their selves and the future. Music programs that capitalize on young people’s strengths and agency (Lerner et al., 2011a; Mueller et al., 2011) and musical engagement and preferences (Bonneville-Roussy et al., 2013), may be particularly suitable for PYD during the challenging middle school years, and we encourage scholars to scrutinize these programs and their potential outcomes.

Macro-time issues (Bronfenbrenner and Morris, 2006) are also worthy of commentary, given their impact on our data and their implications for education. Our study was conducted in the aftermath of a global pandemic that produced devastating effects on schooling, socialization, and mental health. The pandemic reinforced deficit thinking in education, as seen in the increase of narratives of learning loss that place the blame on individuals, children and adolescents alike (Lehman et al., 2021). Several reports have described how students who were socially and/or economically-disadvantaged and those with disabilities were disproportionately affected by the educational inequalities generated and exacerbated by the pandemic (Jandrić and McLaren, 2021). Yet, most responses and solutions proposed to mitigate the educational problems caused by the pandemic have been steeped in a neoliberal educational paradigm, through “econometric forms of analysis” (Jandrić and McLaren, 2021). Following this logic, some schools have reduced or even cut their music programs, replacing them with remedial courses in language arts and mathematics. But as our study suggests, music programs may play a protective role in adolescence. In light of these results, it appears that schools and districts may need to revise approaches to learning that center on deficit models. Attention to the well-established links between youth activity involvement and PYD (Mueller et al., 2011) can assist in the design of programs that could help to mitigate some of the educational losses that were exacerbated by the pandemic. A focus on the development of PYD competencies in schools (see Gomez and Ang, 2007) is also in alignment with the central tenets of positive psychology (Seligman, 2010), including the acquisition of habits that may lead to eudaimonia (Romer and Hansen, 2021). Music may assist in the acquisition of such habits, given its prominent role in the lives of adolescents (Miranda, 2013; Lamont and Hargreaves, 2018). As one participant expressed in a post-study communication with the research team:

Music is the closest thing to a time machine that we will ever have. When we want to transport ourselves into another time or place, we can listen to music or play our instruments, and feel those emotions. Being in the music program has helped me find myself, my peace, and my place in school (7th grade student).

## Limitations and future directions

Despite our recruitment efforts, our study sample was composed predominantly by students who liked and were invested in music, most of which enrolled in the VMSME program. The vast majority of our participants were learning music formally through one or more in-school and/or extracurricular music programs. Additionally, our sample included a large number of students in grade 6, and fewer students in grades 7 and 8. Although this particular sample offered rich information about music learning during the middle school years, future studies could include a larger contingent of students including those in elementary and high school programs and students who are not learning music formally. The adoption of a person-centered, longitudinal, pre- and post-test design, could also help to uncover issues of stability and change in adolescents’ PYD development, as well as shed light on youth developmental trajectories (Good et al., 2011; Oberle et al., 2019). And as our data suggested, it is also vital that future research move beyond heteronormativity, to better represent the identities of today’s students.

Due to privacy issues, we were not able to collect data on music program attendance or school grades, which could have offered additional insights into PYD, HFE and SC in middle school students. By gathering such information, future research could offer a more granular view of student experiences as they relate to musical participation and PYD. Concerning the VMSME program, future studies could also examine the uses of educational apps in relationship to student musical self-efficacy and PYD scores, including its role in the development of Confidence and Competence. Other studies have found these two components to be central to adolescent mental health and wellbeing (e.g., Tomé et al., 2021); hence their relevance in current times. Because individual differences including personality traits have been associated with music learning experiences (see Corrigall et al., 2013), we further suggest that future studies include measures of personality.

Finally, it is important to highlight that our study was conducted in 2022, when the world was still under the impact of a global pandemic. Future studies could offer comparative views of adolescent musical participation and PYD over time. Such information will not only help us situate adolescent development in social and historical perspectives, but will also inform curriculum development and educational policy.

## Data availability statement

The raw data supporting the conclusions of this article will be made available by the authors, without undue reservation.

## Ethics statement

The studies involving human participants were reviewed and approved by USC IRB (protocol # UP21-00906) and the Research Office of the School District where the study took place. Written informed consent to participate in this study was provided by the participants’ legal guardian/next of kin. Participating students also signed assent forms.

## Author contributions

BI designed the study, collected and analyzed data, and worked on manuscript preparation. EC assisted with data collection and analysis, and manuscript preparation. All authors contributed to the article and approved the submitted version.

## Funding

This study was partially funded by the Fender Play Foundation (AWD-00005768) and by USC’s Advancing Scholarship in the Humanities and Social Sciences (ASHSS-2022) grant to BI. The Fender Play Foundation assisted in the liaison with the schools for the purpose of participant recruitment. The Fender Play Foundation grant assisted with research assistant stipend and publication fee.

## Conflict of interest

The authors declare that the research was conducted in the absence of any commercial or financial relationships that could be construed as a potential conflict of interest.

## Publisher’s note

All claims expressed in this article are solely those of the authors and do not necessarily represent those of their affiliated organizations, or those of the publisher, the editors and the reviewers. Any product that may be evaluated in this article, or claim that may be made by its manufacturer, is not guaranteed or endorsed by the publisher.

## Footnotes

^1^ https://measureofamerica.org/human-development/
